# Pilot study: Assessing the effect of continual position monitoring technology on compliance with patient turning protocols

**DOI:** 10.1002/nop2.105

**Published:** 2017-10-26

**Authors:** Suann Cirigliano Schutt, Christine Tarver, Michelle Pezzani

**Affiliations:** ^1^ Program Manager El Camino Hospital Mountain View CA USA; ^2^ Director Medical and Surgical Services El Camino Hospital Mountain View CA USA; ^3^ Hospitalist Palo Alto Medical Foundation Mountain View CA USA

**Keywords:** acute care, compliance, nurse–patient interaction, patient handling, pressure ulcers, technology

## Abstract

**Aim:**

The study aim was to evaluate if continual patient position monitoring, taking into account self‐turns and clinician‐assisted turns, would increase the percentage of time a patient's position changed at least every 2 hr.

**Background:**

While patient turning has clinical benefits, current models to help staff remember to turn patients, such as “turn clocks” and timers, have not resulted in high compliance with turning protocols. In addition, reminders are based on arbitrary 2‐hr windows (such as turning on “even” hours) rather than on individual patient activity, including self‐turns.

**Design:**

This is a first inpatient, non‐randomized, pre‐/postintervention study.

**Methods:**

Data collection occurred from May 2013–February 2014 on a 39‐bed medical unit in a community hospital. Baseline patient turning data were recorded by a sensor; however, the patient data were not displayed at the nurses’ station to establish compliance with the hospital's turning protocol. Postintervention, patient position information was wirelessly displayed on nurses’ station computer monitors in real time. A Student *t* test was used to compare baseline to postintervention “mean time in compliance.”

**Results:**

Data from 138 patients (*N *=* *7,854 hr of monitoring) were collected. The baseline phase yielded 4,322 hr of position monitoring data and the postintervention phase yielded 3,532 hr of data. Statistically significant improvement was demonstrated in the percentage of time a patient's position changed at least every 2 hr from baseline to postintervention.

## 1 INTRODUCTION

### 1.1 Background

Turning patients is one of the most basic nursing care interventions to improve oxygenation (Marklew, 2006), help prevent skin breakdown (Agency for Healthcare Research and Quality (AHRQ), 2014) and improve circulation (Lippincott Advisor, 2015). Prior practices to help remind nursing staff to turn patients included visual reminders, such as a turning clock ((AHRQ), 2014). This regimented type of reminder is potentially ineffective and not patient‐centric, as it does not account for patients’ self‐turning. Other types of prompts have been used—such as signage or auditory alarms—that do not incorporate a patient‐specific approach ((AHRQ), 2014). The 2014 Clinical Practice Guidelines stress the need to consider individual patient needs (such as skin conditions and comfort) when planning repositioning frequency (NPUAP, 2014). However, in deciding any turning protocol, the question of how nursing staff know a patient needs to be turned or has already turned remains, regardless of the standard or physician order for frequency of turning. This study sought to determine if continual position monitoring could increase compliance with a predetermined turn protocol. National Pressure Ulcer Advisory Panel (NPUAP) continues to support the guideline of turning patients every 2 hr to help prevent pressure ulcers and yet acknowledge that this is not always feasible (Black et al., 2011). The desire to reduce pressure ulcers is emphasized in hospitals as it is a nurse sensitive indicator. While there are multiple strategies that can help prevent pressure injuries, including moisture management, frequent turning and nutrition, this study sought to examine the ability to monitor patient movement, as a component of preventing skin breakdown.

A study of patients in elder care homes found that turning protocols of 2 hr compared with a 2‐hr/4‐hr combination did not statistically significantly change the incidence of pressure ulcers (van der Wee, Grypdonck, DeBacquer, & Defloor, 2006). This study supports considering individual patient needs about turning. For the purpose of this study design, the turn frequency protocol against which compliance was measured was 2 hr, as that was the study hospital's nursing standard.

### 1.2 Literature review

Schallom et al. (2005) found accomplishing turning every 2 hr is difficult, noting that although patients had an opportunity for 23 bihourly turns in the observation period, the mean amount of turns was less than half that (Mean = 9.64, range 0–23). Schallom et al. (2005) was limited by the inability to monitor the patients for 24 hr continuously, so activity from 12 midnight to 8 a.m. was not accounted for.

Patient safety about patients in isolation for infectious disease was also reviewed. A systematic review revealed that an increase in adverse events related to failure to care for patients consistently had a negative impact on isolation patient safety (Abad, Fearday, & Safdar, 2010).

While the practice of turning is widely considered essential in inpatient care settings, the ability to monitor and assure the completion of this practice is problematic. NPUAP continues to support the guideline of turning patients every 2 hr to help prevent pressure ulcers yet agree it is not always feasible (Black et al., 2011).

## 2 METHODS

### 2.1 Participants

Eligible participants included adults of all mobility levels age 18 or over who met the inclusion criteria of: (i) hospitalized inpatients expected to remain on unit for at least 12 hr and (ii) able and willing to comply with study procedures.

Exclusion criteria were: (i) cognitive impairment or other mental disability that would prevent the patient (or his/her agent) from providing written informed consent; (ii) known adhesive allergy or sensitivity; (iii) unable to place sensor on patient's anterior torso; (iv) pregnancy or breast feeding; (v) presence of a pacemaker or implanted cardio‐defibrillator (ICD); (vi) patients who refused to have their chest hair clipped if needed for proper sensor placement; and (vii) patients who had participated in another clinical study within the past 30 days.

Participants signed an informed consent form, HIPAA authorization form and California Subject Bill of Rights form. The monitoring system underwent verification and validation testing by the manufacturer, received FDA 510(k) medical device clearance and was registered with clinicaltrials.gov. Once all documents were signed, the patient was enrolled in the study and a sensor was applied. Mobility was not a factor for inclusion or exclusion, as the study hospital has found that pressure injuries have occurred regardless of patients’ ambulation status.

#### 2.1.1 Method of recruitment

Daily unit status reports were generated and reviewed for patients who met the inclusion criteria. A research assistant (RA) interviewed patients who met the inclusion criteria for willingness and the ability to consent to participate in the trial.

#### 2.1.2 Recruitment setting

The study was completed on a 39‐bed adult medical unit at a non‐profit community hospital in Silicon Valley, California. This unit has three nurses’ stations. The patient–nurse ratio was 5:1 and nursing assistants were available on all shifts.

### 2.2 Intervention

#### 2.2.1 Details of intervention

The intervention involved placing a wireless sensor on patients (Leaf Healthcare, Pleasanton, CA, USA) to collect patient position data and evaluate the percentage of time the patient's position was changed within 2 hr. The system consists of a circular 1.5‐inch disposable sensor attached to a film dressing that is adhered to the skin over the patient's sternal area, as well as a computer, server and monitor display. Once fully deployed, the monitor at the nurses’ stations displayed a colour‐coded system used to identify patients who needed to be turned, as follows:

- a “green bar” indicated a patient was turned within the past hour and 45 min;
- a “yellow bar” indicated a patient was complying but required a turn in 15 min or less; and
- a “red bar” indicated a patient was overdue for a turn.

Variables measured. The study was divided into two phases. Patients were categorized as “isolation” or “no isolation.” Additionally, time was captured so the compliance evidence could be examined across a 24‐hr period.

The baseline phase included:

- observation of the system by comparing the patient position transmitted to the computer and visualization of the actual patient in the room; and
- using a sensor to measure each patient's position every 10 s, with the information being transmitted wirelessly to a central server for analysis.

The postintervention phase included:

- using a sensor to measure each patient's position every 10 s, with the information being transmitted wirelessly to a central server and the monitors at the nurses’ stations. Data were downloaded from the server for analysis.

RAs were responsible for:

- consenting patients;
- educating patients about the sensor itself and that their movements would be monitored;
- applying the sensor (which was mounted to an adhesive film dressing) and ensuring sensors were worn continuously; and
- completing case report forms (CRFs) that included patients’ demographic and medical data. CRFs were updated until patients were discharged or transferred off the study unit.

Patients were offered no incentive to participate in the study. Patients were not specifically told the interval for compliance with turns was set at 2 hr. All protected health information (PHI) was transmitted as encrypted data and de‐identified.

During this study, no other changes were made to the organization's turning protocol, including how nurses document a missed patient turn in their notes (i.e. “patient refused”). After the baseline phase, nurses were told that the monitoring system could be used to determine if the hospital's 2‐hr turning standard was being adhered to and that the visualization of which patient needed turning would help them triage their work. Nurses did not change their existing patient rounding practices of patient‐centric care during this study.

### 2.3 Objectives

The study questions were:

- Can the use of a wireless continual position monitoring system increase the percentage of time a patient's position changed within 2 hr for patients on a medical unit?
- Can the use of a wireless continual position monitoring system increase the percentage of time a patient's position changed within 2 hr for patients in isolation?

### 2.4 Outcomes

Primary and secondary outcome measures. Measures used for the study are:

- “turn period,” defined as the “maximum amount of time that can elapse between patient turns”; for this study, the turn period was 2 hr. The turn period automatically reset after an adequate patient turn (either caregiver‐assisted or patient self‐turn). While the system captures all turns, it cannot differentiate between caregiver‐assisted turns or self‐turns as it only monitors movement.
- “turn angle,” defined as “degree of patient rotation in a transverse plane in relation to their vertical axis.” In this study, the turn angle threshold was set to 20 degrees for all patients. Therefore, the turn angle threshold was right side: greater than +20 degrees; back: + 20 to −20 degrees; and left side: less than −20 degrees.
- “decompression time,” defined as the “amount of time a body region (i.e. left, back or right) must be offloaded to be considered fully decompressed.” For this study, a decompression time of 15 min was used.

The monitor at the nurses’ station displays a “green bar” when a turn (self‐turn or caregiver‐assisted) met the defined thresholds for “turn angle” and “decompression time.”

#### 2.4.1 Data collection methods

Once consent was obtained, the patients were enrolled in the study and an RA applied a sensor to each patient's sternal area and initiated data transmission.

Data for the study were collected on a centralized server that captured continual movements of all study patients every 10 s. Raw data from the patient sensors were imported into a custom reporting tool and analysed using Microsoft Excel^™^. Individual position data were then grouped and analysed in 1‐hr increments.

#### 2.4.2 Validated instruments

The sensor module used a micro‐electromechanical system (MEMS) accelerometer to measure each patient's position, expressed to the user as a roll, tilt or upright angle. MEMS accelerometers have been used for over 30 years in several performance and safety‐critical applications, including aeronautic navigation, hard disk protection mechanisms and automobile airbags. The accuracy of the sensor module is monitored throughout its design cycle using an angle gauge professionally calibrated by a third‐party calibration company using National Institute of Standards and Technology traceable calibration equipment. Additionally, each sensor received a full functional validation test before being packaged for use. This functional test included rotating the sensor along each of its axes to ensure proper functionality of its accelerometer and checks to ensure proper communication of its orientation data. The baseline phase of visual matching of the patient position and computer monitor/server display of patient position (right, back and left) was not kept in the testing documentation. Additional validation testing is performed for each sensor software release and consists of monitoring a batch of sensors in a simulated environment reporting at 10× the normal rate until end‐of‐life.

### 2.5 Sample size

A target of 63 patients was selected based on achieving a 95% confidence interval, assuming a standard deviation of 0.5 and a margin of error of 12.3 percentage points. The following formula was used to calculate the estimated number of patients:

$$n=\;\frac{\left(Z^{2}\;\times \;sd^{2}\right)}{e^{2}}$$

(*n* = target sample size, *sd* = standard deviation, *e* = margin of error).

Due to patient turnover on the unit, the baseline phase and postintervention phase patients were not the same patients.

### 2.6 Assignment method

All patients were considered for this convenience sample study. Any patient who met the inclusion criteria and agreed to participate was included.

### 2.7 Blinding

As the patients needed to have a sensor applied, they were not blinded to the study. In the baseline phase, nurses were not specifically instructed as to what the sensor was monitoring.

### 2.8 Unit of analysis

Data from the continual position monitoring sensor were transmitted every 10 s.

### 2.9 Statistical methods

A *t* test was used to evaluate the difference in turn protocol compliance between the baseline phase sample and the postintervention sample. “Compliance” was not measured as a categorical variable but rather as a per cent of variability, ranging anywhere from 0% to 100% and therefore was a parametric variable. As the true standard deviation of the population was unknown and the sample size was expected to be relatively small, a t‐score was selected over a z‐score.

The turning protocol compliance rate for each patient was calculated as described below. The individual turn protocol compliance for patients in the baseline and postintervention phases was calculated by dividing the sum of the “total time a patient was considered compliant with the turn protocol” by “total monitored time.” The average turn protocol compliance for all patients in the baseline and postintervention phases was calculated by dividing the “sum of the total time each patient in the phase was compliant with the turn protocol” by the “total monitored time.” A patient was considered in compliance if the sustained time on a given side (left, back or right) was less than the assigned turn period. During the postintervention phase, individual patient compliance was communicated to the nursing staff using a three‐colour system. Patients who displayed green or yellow were compliant. Patients who displayed red were non‐compliant.

All compliance calculations incorporated a 15‐min grace period in the turn period. As all patients were on a 120‐min turning protocol, there was no impact on compliance metrics until a patient accumulated 135 min on one side. The grace period was incorporated into both the pre‐intervention and postintervention compliance calculations. However, it was not considered when displaying the compliance state of patients during the intervention phase and nursing staff were not made aware of the grace period.

To identify trends and variability in the compliance rate, “adherence to the turn protocol over time” was also analysed. Each phase was segmented into 100‐hr monitoring intervals, which can consist of data from one or more patients. The percentage of time patients were 100% compliant for a given hour was then calculated for each consecutive block of 100 patient monitoring hours. The “turn protocol adherence” was calculated by taking the sum of hours with “100% turn protocol compliance” and dividing by “100 hr of monitoring time.”

To monitor for changes in turning compliance throughout the day (and therefore by shift), the average compliance over the course of the study was calculated in 1‐hr time blocks for each hour of the day. The average hourly compliance was calculated by summing the total number of hours patients were in compliance for each 1‐hr time block divided by the total number of patient hours monitored for that time block. The “average hourly turn protocol compliance” was calculated by dividing the sum of “time in compliance with turn protocol per patient for a given hour interval each day” by “total time monitored per patient for a given hour interval each day.”

### 2.10 Ethical considerations

The study was conducted according to the relevant and applicable regulations under U.S. Food and Drug Administration 21 CFR Part 812 and 21 CFR Parts 50 and 56 and in accordance with local laws and regulations. Written informed consent was obtained from each patient prior to participation.

## 3 RESULTS

### 3.1 Participant flow

**Figure 1:**
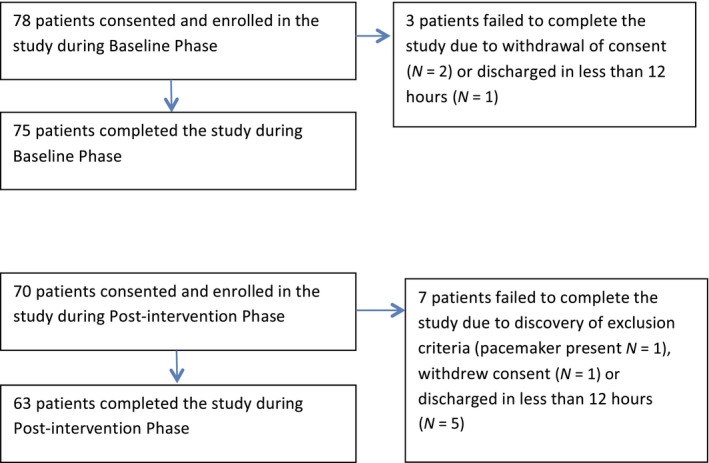
Participant flow for baseline phase and postintervention phase

All patients admitted to the unit during the time of the study were screened by the study coordinator for obvious exclusion criteria. Those with no recognizable exclusions based on the chart review were approached for consent. No records were kept on any patient who appeared to qualify based on the chart review or on those who were approached for consent but did not enroll. See Figure 1 for participant flow.

Protocol deviations in the study included: lack of hair clipping before applying the sensor (*N *=* *4); patient discharged with sensor still applied, necessitating phone call follow‐up to check adverse events (*N *=* *2); sensor fell off and was replaced by RN (*N *=* *3); and sensor applied to patient who was later found to have a pacemaker (*N *=* *1). The pacemaker patient was eliminated from the study as the sensor was removed within 1 hr; there were no adverse events.

### 3.2 Recruitment

The project was submitted for review to the Institutional Review Board of the study hospital and was approved. Next, the wireless infrastructure/technology was tested and installed. The first patient was enrolled in May 2013 and the last was enrolled in February 2014. Data analysis commenced soon thereafter.

### 3.3 Baseline data

Data for 138 patients across both phases were included in the analysis, representing 7,854 hr of position data. The baseline phase sample (*N *=* *75) consisted of a different set of patients than did the postintervention phase sample (*N *=* *63). Demographic information is displayed in Table 1. There were five isolation patients in each sample.

**Table 1:** Demographic data

	Baseline phase sample	Postintervention phase sample
Number of patients enrolled	78	70
Number of patients completing study	75	63
Total monitoring hours	4,445	3,537
Average age	58 (range 20–95)	63 (range 23–97)
Per cent female	47%	56%
Average Braden score	20.8 (range 11–23)	19.9 (range 11–23)
Number of isolation patients	5	5
Average age of isolation patients	56.6 (range 26–86)	58 (range 42–68)
Per cent female of isolation patients	60%	40%
Average Braden Score of isolation patients	19.7 (range 13.8–21.6)	21.2 (range 20.8–22)
Total monitoring hours for isolation patients	377.4	301.6

A chi‐square test for independence was used to evaluate differences in gender between the baseline and postintervention samples. A *p*‐value of .292 was obtained, indicating no statistical difference in gender between the two samples.

The average Braden scores for the baseline and postintervention sample populations were 20.8 and 19.9 respectively. *p *=* *.016 was obtained using a two‐tailed *t* test to evaluate the distribution of Braden scores between the two groups, indicating the postintervention group had a lower average Braden score by 0.9; the difference was significant (*p *<* *.05).

### 3.4 Numbers analysed

In both groups, patient data were collected and analysed continuously throughout the entire time a patient was wearing the sensor (the “wear time” of the patient sensor).

### 3.5 Outcomes and estimation

The *t* test results indicate that using a wireless continual position monitoring system statistically significantly increased the percentage of time a patient's position was changed within 2 hr (64%–98%, *p *<* *.001). T test results also revealed the use of a wireless continual position monitoring system statistically significantly increased the percentage of time a patient's position was changed within 2 hr for patients in isolation (48%–99%, *p *=* *.030).

**Figure 2:**
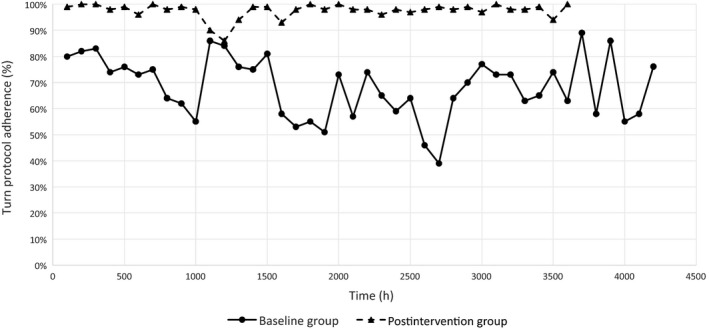
Turn protocol adherence comparison: Baseline vs. Postintervention

Figure 2 displays the turn protocol adherence comparison—baseline vs. postintervention. T test results revealed lower baseline phase turn adherence of 39%–89% (average 64%) compared with postintervention phase turn adherence of 86%–100% (Mean 98%). The improvement in turn protocol adherence was statistically significant (*p *<* *.001).

There are instances when required turns cannot be performed (i.e. clinical circumstances, patient refusal, patient being off unit). If the caregiver documented a valid reason a required turn could not be performed (captured through chart audit of nurses’ notes by an RA), the missed turn did not have a negative impact on compliance calculations.

### 3.6 Ancillary analyses

**Figure 3:**
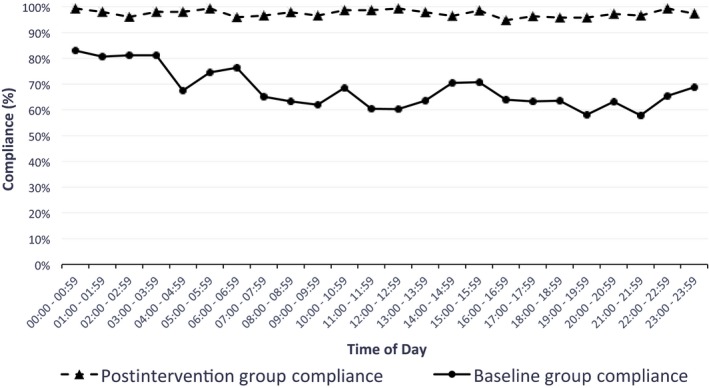
Average hourly turning compliance

Figure 3 displays the average hourly turning compliance, reflecting cyclical changes that occur in turn adherence over 24 hr. After implementation of the monitoring system, cyclical variation decreased and the compliance rate was higher at all hours of the day.

### 3.7 Adverse events

During the study, a small number of patients (*N *=* *2) experienced mild temporary skin irritation believed to be related to the adhesive on the film dressing used to attach the sensor to the sternal area. The RAs, who were Registered Nurses, made the initial assessment of skin irritation. All incidences of skin irritation (*N *=* *2) were reported to the physician principle investigator. Both patients with skin irritation were followed and the irritation resolved without additional intervention. As these two cases of mild skin irritation self‐resolved, this was not felt to be a barrier to product use.

The film dressing for adherence of the patient sensor was selected because it is a common dressing used in the study hospital for IV dressings without any significant report of patient skin irritation. The literature review revealed the prevalence of adverse skin reactions is “unknown, as official figures are unavailable and where they do exist are thought to be unreliable” (Conway & Whettam, 2002). There was one study where film dressings were compared with pressure dressings for postpercutaneous transluminal coronary angiography patients; one patient in a sample of 35 was found to have skin irritation (McIe, Pettite, Pride, Leeper, & Ostrow, 2009). This present study also found very low incidence of skin irritation with film dressings.

## 4 DISCUSSION

### 4.1 Interpretation

For the first time, a continual position monitoring system was used to accurately and objectively measure compliance with patient turning protocols. The baseline compliance at the study hospital, as objectively determined by continuous position monitoring, was consistent with previous studies (Tucker, 2009 and Voz, Williams, & Wilson, 2011). The baseline phase sample benefitted from the electronic health record worklist reminders to turn patients, yet still had compliance of 64% with 2‐hr turning. This finding demonstrates that electronic reminders did not yield a high compliance with turning protocols at the study hospital.

Traditionally, turning protocols have taken a “one size fits all” approach that is not patient‐centred. Continual position monitoring technology may enable nursing staff to better individualize patient care in response to the national push towards a patient‐centred healthcare model. Given that the system continually tracks patient movement, credit is given for any adequate patient self‐turns; thus, the system may prevent unnecessary work and patient disruptions and improve staff efficiency. When nursing staff noticed on the monitor that a patient turn was required (i.e. the turn “bar” had become red), a notification was provided to the primary nurse and/or nursing assistant so the patient could be turned as soon as possible. An administrative support person (“unit clerk”) often watched the monitor at the main nursing station near where she answered phones and call lights and was able to inform the nurse or nursing assistant that a turn was needed. An informal, non‐scientific questionnaire was given to nursing staff during the baseline phase and again in the postintervention phase for anonymous feedback on the continual position monitoring system. In the postintervention phase questionnaire, 87% (41 of 47) of the nursing staff felt the continual position monitoring system was helpful. The nurses said the system helped them prioritize patient care and avoid unnecessary tasks.

By turning patients every 2 hr on a “set schedule” (i.e. turn on even hours), there is a risk of turning a patient to a position they had just recently turned away from themselves. The continual position monitoring system allows nursing staff to know the length of time in a position and to plan care accordingly.

There were limitations in terms of the sample size and sample equality of the two phases. The data collection period for the baseline phase was approximately four times as long as the postintervention phase due to slower enrolment rates in the baseline phase. There was only one RA during the baseline phase, thus causing slower enrolment. During the postintervention phase, additional RAs were hired to speed the enrolment process. While the number of patients was similar between the two phases, the data for the baseline group were collected over a period of 18 weeks and that for the intervention group were collected over a period of 4 weeks. Therefore, the baseline phase had fewer patients monitored per day than the postintervention phase.

The methodology chosen to calculate compliance was developed by the research team because there was no standard method available. Although there could be other methods, the research team believed the chosen definition was most consistent with the practical expectations of what constitutes compliance.

This study was non‐randomized and non‐blinded. Given that the intervention under investigation required a clinical process change and the adoption of new procedures, it was not possible to randomize patients or staff. Although the lack of randomization makes it harder to rule out confounding variables, efforts were made to ensure no other policies or process changes were introduced during the study period. About blinding, due to the baseline and postintervention nature of the study, it was not possible to blind nursing staff.

In terms of assessing the baseline turning protocol compliance, efforts were made to minimize the introduction of observer effect bias. Although the nursing staff was not explicitly made aware of the nature of the study, it is difficult to estimate the degree to which the Hawthorne effect may have artificially increased our baseline turning compliance.

The primary outcome measure of this study was to determine if continual position monitoring could increase the percentage of time a patient's position changed within 2 hr. It was believed that the increase was achieved by taking into account the patient's own abilities for self‐turning and clinical turning, thereby personalizing while prioritizing patients’ care needs.

Goldhill, Badacsonyi, Goldhill, and Waldmann (2008) supported the need to develop a better understanding of patient turning and the associated clinical benefits. The authors found the average time between turns was 4.85 hr (*N *=* *393) with no statistical difference in a variety of demographics, including gender, age, intubation status and sedation. They also reported that 42% of patients were turned in 2 hr or less and that turning patients regularly can have various potential benefits, including decreased muscle wasting, improved lung capacity and decreased pressure‐related skin issues (Goldhill et al., 2008).

Future studies should include replication with a larger sample size to evaluate patient care processes and clinical outcomes, such as pressure ulcers, clot formation and respiratory status, beyond simply turning compliance. While no patients who were part of this study developed a pressure ulcer while participating, a larger sample size would be better to evaluate the incidence of pressure ulcers when using the monitoring system. A larger sample size could also increase the number of isolation patients studied to evaluate the turning compliance changes for that patient population. Additional studies could examine variables of Braden category score for mobility and activity in comparison to self‐turning. This could help determine if patients who are highly mobile and active also self‐turn while in bed. Studies outside of California would also be beneficial due to percentage of RN staffing variability, beyond the California state‐mandated nurse‐to‐patient ratios which leads to a higher percentage of RNs on medical‐surgical units.

There was a serendipitous finding in the study about a morbidly obese patient who needed more pillows to stay in the side‐lying position than the nursing staff had originally placed. This was noted due to the monitoring system not crediting an offloading turn based on the 20% angle turn system requirement. The patient had “sunk back” so far into the original pillows that the 20% turn change had not been maintained and the system “bar” returned to red. This finding suggests knowledge could be gained through future study of continual positioning monitoring of morbidly obese patients.

Future studies could also include patient experience, nursing satisfaction with the system and more evaluation of the challenges of accurately offloading data for extremely obese patients.

### 4.2 Generalizability

This study was conducted on one medical unit in a community hospital in Silicon Valley, California, thus limiting its generalizability. Future studies would need to replicate the study in different settings, such as academic institutions and in different states where the nurse‐to‐patient ratio is not mandated. The study should also be replicated with different patient populations, such as intensive care patients, postsurgical patients and/or long‐term care patients.

### 4.3 Overall evidence

The display of patient movement data at the nurses’ stations enabled nurses to identify patients who were self‐turning and patients who were in need of assisted turns. The enhanced ability to understand patient positioning could have potential benefits in terms of patient safety, the prevention of falls and pressure ulcers and more accurate triaging of patient care needs with regard to patient turning.

## CONFLICT OF INTEREST

No conflict of interest has been declared by the authors.

## AUTHOR CONTRIBUTIONS

All authors agreed on the final version and meet at least one of the following criteria [recommended by the ICMJE (http://www.icmje.org/recommendations/)]:

- substantial contributions to conception and design, acquisition of data, or analysis and interpretation of data; and
- drafting the article or revising it critically for important intellectual content.

## References

[nop2105-bib-0001] Abad, C. , Fearday, A. , & Safdar, N. (2010). Adverse effects of isolation in hospitalized patients: A systematic review of isolation. Journal of Hospital Infection, 76(2), 97–102. 20619929 10.1016/j.jhin.2010.04.027 PMC7114657

[nop2105-bib-0002] Agency for Healthcare Research and Quality (AHRQ) . (2014). Preventing pressure ulcers in hospitals: A toolkit for improving quality of care. Available from http://www.ahrq.gov/professionals/systems/hospital/pressureulcertoolkit/index.html. [last accessed 3 January 2016].

[nop2105-bib-0003] Black, J. M. , Edsberg, L. E. , Baharestani, M. E. , Langemo, D. , Goldberg, M. , McNichol, L. , Cuddigan, J. (2011). Pressure ulcers: Avoidable or unavoidable? Ostomy Wound Management, 2011, 57, 24–37. 21350270

[nop2105-bib-0004] Conway, J. , & Whettam, J. (2002). Adverse reactions to wound dressings. Nursing Standard, 16(44), 52–60. 10.7748/ns2002.07.16.44.52.c3232 12219515

[nop2105-bib-0005] Goldhill, D. R. , Badacsonyi, A. , Goldhill, A. A. , & Waldmann, C. (2008). A prospective observational study of ICU patient position and frequency of turning. Anaesthesia: Journal of the Association of Anaesthetists of Great Britain and Ireland, 63, 509–515, https://doi.org/10.1111/j.1365-2044-2007.0543.x 10.1111/j.1365-2044.2007.05431.x 18412649

[nop2105-bib-0006] Lippincott Advisor . (2015). Diseases and conditions: Turning. Available from: http://advisor.lww.com/lna/home.do?setCookie=1680. [last accessed 3 October 2017].

[nop2105-bib-0007] Marklew, A. (2006). Body positioning and its effects on oxygenation: A literature review. Nursing Critical Care, 11(1), 16–22. 10.1111/j.1362-1017.2006.00141.x 16471294

[nop2105-bib-0008] McIe, S. , Pettite, T. , Pride, L. , Leeper, D. , & Ostrow, C. L. (2009). Transparent film dressing vs pressure dressing after percutaneous transluminal coronary angiography. American Journal of Critical‐Care Nurses, 18(1), 14–19. https://doi.org/10.4037/ajcc2009949 10.4037/ajcc2009949 19116400

[nop2105-bib-0009] National Pressure Ulcer Advisory Panel, European Pressure Ulcer Advisory Panel and Pan Pacific Pressure Injury Alliance (NPUAP) . (2014). Prevention and treatment of pressure ulcers: Clinical practice guideline. Haesler E. (Ed.). Osborne Park, WA: Cambridge Media.

[nop2105-bib-0010] Schallom, L. , Methany, N. A. , Stewart, J. , Schnelker, R. , Ludwig, J. , Sherman, G. , & Taylor, P. (2005). Effect of frequency of manual turning on pneumonia. American Journal of Critical Care, 14(6), 476–478. 16249585

[nop2105-bib-0011] Tucker, M. E. (2009). Repositioning guidelines often not followed. Caring for the Ages, 10, 15 Available from: http://www.caringfortheages.com/article/S1526-4114(09)60100-1/pdf.

[nop2105-bib-0012] Voz, A. , Williams, C. , & Wilson, M. (2011). Who is turning the patients?: A survey study. Journal of Wound Ostomy Continence Nursing, 38(4), 413–418. https://doi.org/10.1097/WON.0b013e318220b6ec 10.1097/WON.0b013e318220b6ec 21747258

[nop2105-bib-0013] van der Wee, K. , Grypdonck, M. H. F. , DeBacquer, D. , & Defloor, T. (2006). Effectiveness of turning with unequal time intervals on the incidence of pressure ulcer lesions. Journal of Advanced Nursing, 57, 59–68. 10.1111/j.1365-2648.2006.04060.x 17184374

